# Treatment pattern and clinical outcomes in portopulmonary hypertension: A database study in Japan

**DOI:** 10.1002/jgh3.12820

**Published:** 2022-10-13

**Authors:** Masanori Atsukawa, Masashi Takano, Junichi Omura

**Affiliations:** ^1^ Division of Gastroenterology and Hepatology Nippon Medical School Tokyo Japan; ^2^ Medical Affairs Division Janssen Pharmaceutical K.K. Tokyo Japan

**Keywords:** database, portal hypertension, portopulmonary hypertension, pulmonary arterial hypertension, treatment pattern

## Abstract

**Background and Aim:**

Portopulmonary hypertension (PoPH) is a complication associated with portal hypertension. Since the epidemiological study in Japan was limited, this study aimed to investigate the characteristics, treatment patterns, and prognosis of PoPH patients in real‐world data.

**Methods:**

The characteristics and treatment patterns of PoPH (*n* = 386) and portal hypertension without pulmonary arterial hypertension (portal hypertension w/o PAH) (*n* = 96 463) were analyzed based on the Medical Data Vision (MDV) dataset from April 2008 to September 2020. Survival‐time analyses of emergency hospitalization and mortality were also conducted between matched pair cohorts of PoPH (*n* = 210) and portal hypertension w/o PAH (*n* = 840).

**Results:**

Among 386 PoPH patients, the Child–Pugh classification of PoPH group comprised patients with Class A (59 [15.3%]), B (109 [28.2%]), and C (42 [10.9%]), and missing (176 [45.6%]). Regarding the feature of PoPH group, the proportion of primary biliary cholangitis (PBC) (13.7%) and splenomegaly (9.8%) was higher compared with portal hypertension w/o PAH group. The survival time of all‐cause hospitalization in PoPH group was shorter than portal hypertension w/o PAH group in matched pair cohort (*P* < 0.001 by log‐rank test). Of PoPH patients, the frequency of PAH‐specific medicine usage within 90 days was monotherapy of 79 patients (20.5%), combination therapy of 64 patients (16.6%), and PAH‐specific medicine usage of 243 patients (63.0%).

**Conclusion:**

This was the first study demonstrating that high proportion of PBC and splenomegaly and a greater risk of hospitalization were observed in PoPH patients based on the analysis using administrative claim database.

## Graphical abstract

This was the first study demonstrating that high proportion of PBC and splenomegaly and a greater risk of hospitalization were observed in PoPH patients based on the analysis using administrative claim database. The findings of this study may lead to the improvement of PoPH disease awareness and medical care in the future.

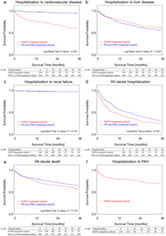

## Introduction

Portopulmonary hypertension (PoPH), a progressive disease having a poor prognosis, is the pulmonary arterial hypertension (PAH) associated with portal hypertension. Among the patients with portal hypertension, 1.1–6.3% were reported to have PoPH.^1^, ^2^, ^3^

As of today, the standard treatment of PoPH has not been established. According to the PAH guidelines published by the European Society of Cardiology (ESC) and the European Respiratory Society (ERS) in 2015, the treatment of PoPH was advised to follow the Group 1 of PAH.^4^ Several studies have shown the efficacy of pulmonary vasodilators in PoPH.^5^, ^6^, ^7^ Recently, the efficacy of Macitentan was reported based on changes in pulmonary artery pressure (PVR)^8^ and based on the reduction of the perioperative mortality risk in patients scheduled for liver transplantation.^9^

Epidemiological results of PoPH have been reported in Europe and the United States,^2^, ^10^, ^11^ but only a few epidemiological studies with limited information on PoPH from Japan were published.^3^, ^12^ Therefore, the treatment of PoPH was largely based on the limited evidence and referred to previously classified PAH.^13^

The aim of this study was to investigate the real‐world patient characteristics, treatment patterns, prognosis, and causes of death using the administrative claim database in Japan to better understand the disease epidemiology and improve the medical care for PoPH patients.

## Methods

### Data source

This was a retrospective database study using the administrative claims database, which included information from medical institutions employing Diagnosis Procedure Combination (DPC) system and was provided by Medical Data Vision Co., Ltd. (Tokyo, Japan) (MDV). In this study, data from patients who had a diagnosis (ICD‐10:K70‐77) of hepatic diseases between April 2008 and September 2020 were extracted regardless of gender and clinical settings (inpatient or outpatient clinics). Data collected by MDV were anonymized after obtaining secondary use permission at the contracted hospitals.

### Study design and sample

**Figure 1:**
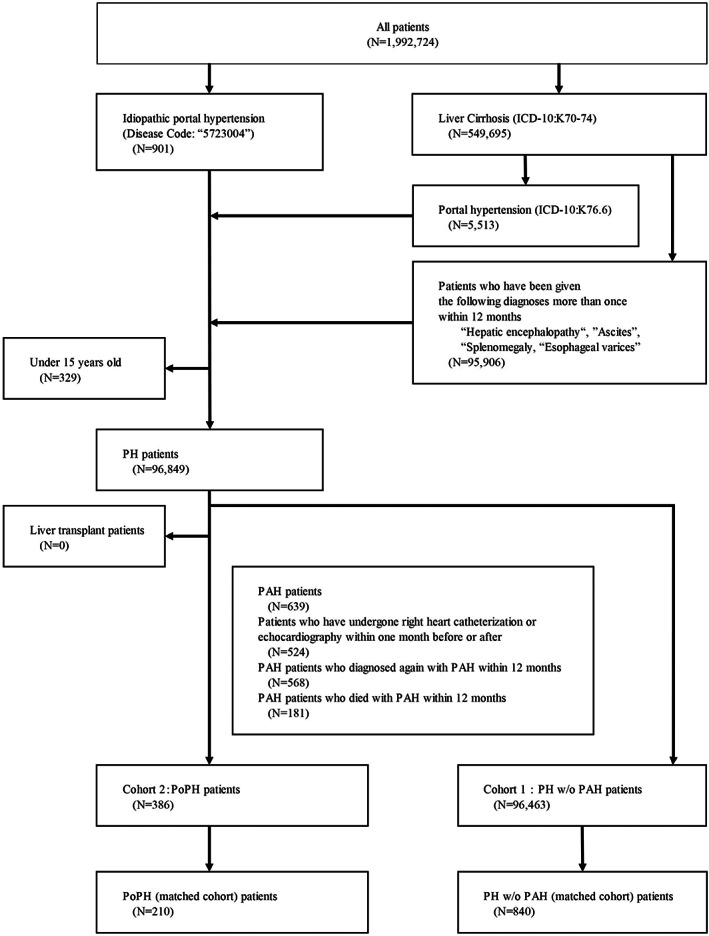
Flowchart showing the process of patient selection in the study. Total number of patients in the target period was 96 463 in the portal hypertension w/o PAH group (Cohort 1) and 386 in the PoPH group (Cohort 2). After propensity score matching, information from 210 patients in the PoPH group and 840 patients in the portal hypertension w/o PAH group was extracted.

This study included patient populations of portal hypertension without pulmonary arterial hypertension (portal hypertension w/o PAH) and PoPH (Fig. 1). Patient data recording at least one injury and disease name of the targeted disease during the data coverage period were retrieved.

The Index date for each patient was the diagnosis date of portal hypertension w/o PAH or PoPH. Eligibility descriptions of patient populations in portal hypertension w/o PAH and PoPH groups are shown in Figure 1 (see Tables S1 and S2, Supporting information). The study was conducted in accordance with the ethical principles of the Declaration of Helsinki and was consistent with the Japanese Ethical Guidelines for Medical and Biological Research Involving Human Subjects. Ethical review and informed consent were not required, because the study used retrospective de‐identified data.

### Variables

#### Primary outcome

As primary outcomes, demographic characteristics of patients with portal hypertension w/o PAH and PoPH are shown in Table 1. The treatment patterns of both portal hypertension w/o PAH and PoPH groups were summarized and divided into four time periods of 90 days from Index date (within 90 days), 91–365 days (within 1 year), 366–730 days (within the second year), and 731–1095 days (within the third year). Drugs for the treatment of liver diseases and pulmonary vasodilators are shown in Table S3, Supporting information. Pulmonary vasodilators were included in both categories of monotherapy and combination therapy as tabulated. Frequency calculation of pulmonary vasodilators in PoPH group with subdivision of drug generic names was also performed.

**Table 1:** Patient characteristics summary

	PH w/o PAH	PoPH	PH w/o PAH (matched cohort)	PoPH (matched cohort)
	*n* (%)	*n* (%)	*n* (%)	*n* (%)
Total	96 463	386	840	210
Age (years)^†^				
*n*	96 463	386	840	210
Mean (SD)	67.8 (12.9)	65.6 (16.4)	68.5 (12.9)	66.6 (15.0)
Min–med–max	15–69.0–100	15–69.0–94	19–71.0–94	18–69.0–89
Age category				
16–30	741 (0.8)	19 (4.9)	4 (0.5)	7 (3.3)
31–40	2083 (2.2)	13 (3.4)	18 (2.1)	7 (3.3)
41–50	7264 (7.5)	30 (7.8)	62 (7.4)	14 (6.7)
51–60	14 864 (15.4)	56 (14.5)	141 (16.8)	33 (15.7)
61–70	27 039 (28.0)	87 (22.5)	193 (23.0)	53 (25.2)
71–80	28 888 (29.9)	115 (29.8)	256 (30.5)	57 (27.1)
≥81	15 563 (16.1)	64 (16.6)	166 (19.8)	39 (18.6)
Sex^†^				
Male	59 876 (62.1)	156 (40.4)	275 (32.7)	75 (35.7)
Female	36 587 (37.9)	230 (59.6)	565 (67.3)	135 (64.3)
Child–Pugh classification^†^				
A	13 099 (13.6)	59 (15.3)	221 (26.3)	59 (28.1)
B	23 150 (24.0)	109 (28.2)	446 (53.1)	109 (51.9)
C	19 476 (20.2)	42 (10.9)	173 (20.6)	42 (20.0)
Missing	40 738 (42.2)	176 (45.6)	0 (0.0)	0 (0.0)
NYHA classification				
I	150 (0.2)	15 (3.9)	2 (0.2)	10 (4.8)
II	235 (0.2)	22 (5.7)	1 (0.1)	17 (8.1)
III	261 (0.3)	32 (8.3)	6 (0.7)	21 (10.0)
IV	190 (0.2)	22 (5.7)	0 (0.0)	11 (5.2)
Missing	95 627 (99.1)	295 (76.4)	831 (98.9)	151 (71.9)
Concurrent disease				
Idiopathic portal hypertension	811 (0.8)	12 (3.1)	10 (1.2)	5 (2.4)
Liver cancer^†^	53 158 (55.1)	167 (43.3)	399 (47.5)	103 (49.0)
Hepatopulmonary syndrome	89 (0.1)	10 (2.6)	4 (0.5)	8 (3.8)
Ascites^†^	36 494 (37.8)	135 (35.0)	253 (30.1)	65 (31.0)
Esophageal varices^†^	41 950 (43.5)	152 (39.4)	448 (53.3)	112 (53.3)
Esophageal varices (with bleeding)^†^	7401 (7.7)	22 (5.7)	100 (11.9)	20 (9.5)
Esophageal varices (without bleeding)^†^	38 071 (39.5)	146 (37.8)	403 (48.0)	107 (51.0)
Splenomegaly	3755 (3.9)	38 (9.8)	22 (2.6)	14 (6.7)
Hepatic encephalopathy^†^	44 426 (46.1)	173 (44.8)	448 (53.3)	104 (49.5)
Etiology of liver cirrhosis^†^				
Viral (Type B/C)	32 515 (33.7)	94 (24.4)	236 (28.1)	57 (27.1)
Metabolic (alcoholic /non‐alcoholic)	28 432 (29.5)	79 (20.5)	155 (18.5)	42 (20.0)
AIH (autoimmune hepatitis)	4276 (4.4)	26 (6.7)	47 (5.6)	16 (7.6)
PBC (primary biliary cholangitis)	5821 (6.0)	53 (13.7)	139 (16.5)	33 (15.7)
Others	25 419 (26.4)	134 (34.7)	263 (31.3)	62 (29.5)

^†^ Covariates.

PH w/o PAH, portal hypertension without pulmonary arterial hypertension; PoPH, portopulmonary hypertension; SD, standard deviation.

#### Secondary outcome

For secondary outcomes, the survival‐time analysis of the following events, including emergency hospitalization (hereafter referred to hospitalization), death of patients in PoPH group (after matched), and portal hypertension w/o PAH group (after matched), were performed and categorized as follows: (i) Hospitalizations related to cardiovascular diseases; (ii) hospitalizations related to liver diseases; (iii) Hospitalizations related to renal failure; (iv) hospitalizations of all cause; (v) death of all cause. For PoPH group, survival time analysis was also performed for hospitalization due to PAH. Survival‐time analysis of indicated items was also performed for Child–Pugh classification.

The following analyses were conducted as additional information. (i) Analysis of patient characteristics and treatment patterns for match‐paired cohort in portal hypertension w/o PAH and PoPH groups. (ii) Calculation of incidence of complications per observation in indicated period (chronic lung disease, rheumatic disease, diabetes mellitus, renal disease, and malignancy) in portal hypertension w/o PAH and PoPH groups. (iii) Within PoPH group, calculation of the percentage of injury, and disease names draining the most medical resources at the time of death record for evaluating the potential cause of death.

### Statistical analysis

Continuous variables were summarized using sample size, mean, standard deviation, minimum, median, maximum, and interquartile range. Discrete variables were presented as percentage of the sample size and the number of patients included in the analysis as needed.

The survival‐time analysis calculated the number of days from Index date to the date of events or censoring. Kaplan–Meier method was employed for the survival analysis of patients with one to four recorded hospitalizations or deaths as event case and patients without events as censoring case. Log‐rank test was also performed between PoPH and portal hypertension w/o PAH group. For PAH‐related hospitalizations, the judgments of physicians were made based on the medical information recorded in the discharge summary and the duration of hospitalization.

Propensity score analyses were conducted by calculating and matching propensity score with the cohort as the objective variable and covariates as the explanatory variables. Covariates included age, sex, etiology of liver disease (viral, metabolic, autoimmune hepatitis [AIH], primary biliary cholangitis [PBC], and others), Child–Pugh class, and presence of symptoms of non‐metabolic cirrhosis (ascites, esophageal varices, hepatic encephalopathy, and liver cancer).^10^, ^14^, ^15^ Propensity score matching was analyzed using PSMATCH procedure of SAS. All statistical analyses were performed using SAS® ver.9.4 (SAS Institute Inc., Cary, NC, USA).

## Results

### Patient characteristics

Data processing procedure and patient characteristics are shown in Figure 1, Table 1, and Table S4, Supporting information. There were 96 849 patients in portal hypertension group, of whom 96 463 patients were in portal hypertension w/o PAH (mean age ± SD: 67.8 ± 12.9, 62.1% of male) and 386 in PoPH group (mean age ± SD: 65.6 ± 16.4, 40.4% of male). Among 386 PoPH patients, the characteristics of PoPH patients were as follows: (i) In terms of age, the percentage of patients aged 61 or older was 68.9% (266 patients); (ii) in Child–Pugh class, PoPH group had more B classifications (109, 28.2%); (iii) regarding comorbidities, for PoPH group, the proportions of splenomegaly (38, 9.8%) and hepatopulmonary syndrome (10, 2.6%) were higher than the proportions of splenomegaly (3755, 3.9%) and hepatopulmonary syndrome (89, 0.1%) in portal hypertension w/o PAH group (Table 1). Of PoPH patients, the most common etiology of cirrhosis was viral hepatitis (94, 24.4%), followed by metabolic cause (79, 20.5%). PoPH group had a higher percentage of PBC (53, 13.7%) than portal hypertension w/o PAH group (5821, 6.0%) (Table 1).

### Time‐to‐event survival analysis

**Figure 2:**
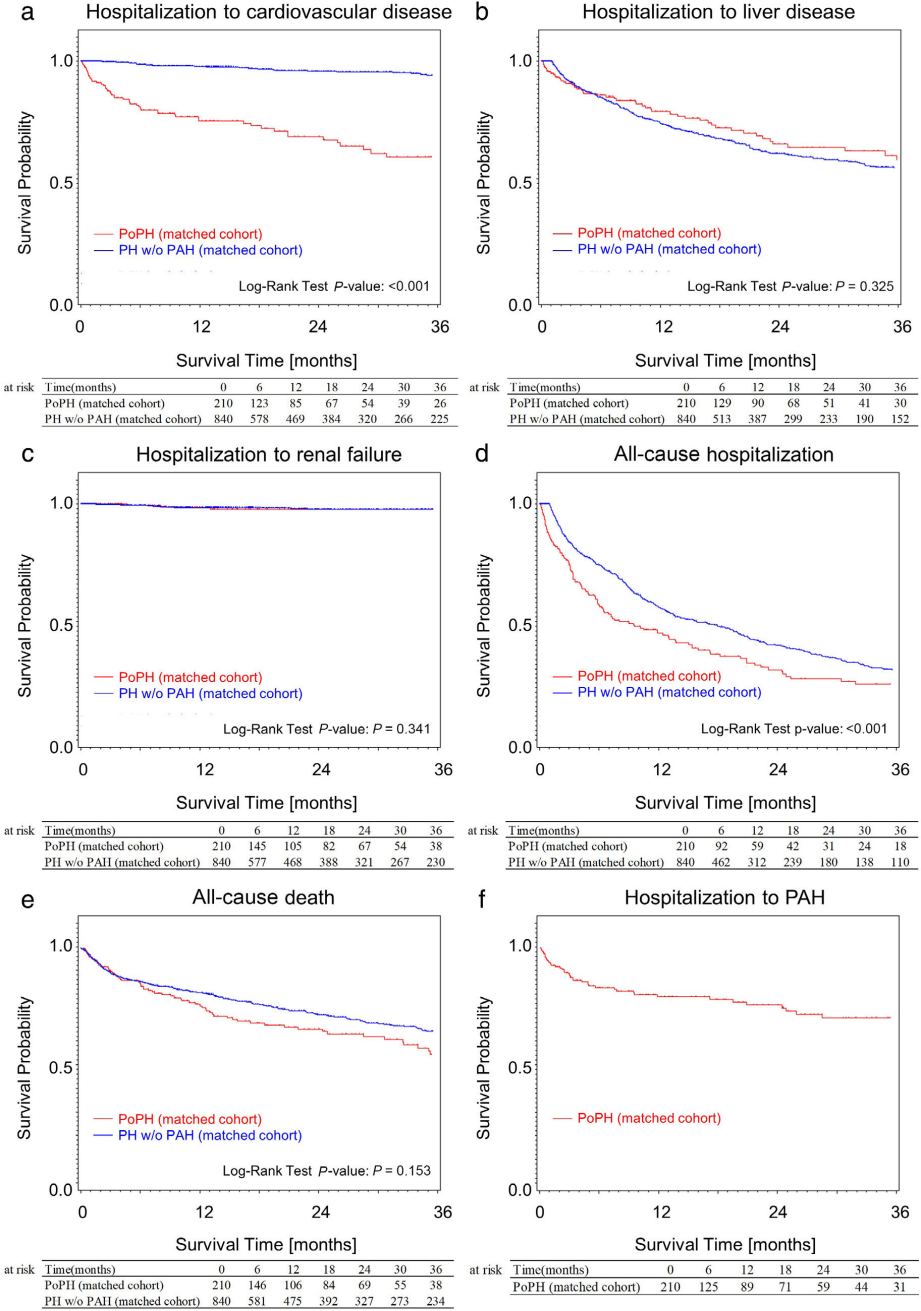
The survival‐time analysis of the following six events defined in this study is shown; (a) emergency hospitalization related to cardiovascular disease, (b) emergency hospitalization related to liver disease, (c) emergency hospitalization related to renal failure, (d) emergency hospitalization of all cause, (e) death related time to all causes, and (f) emergency hospitalization related to PAH time to hospitalization. Analysis of PoPH 210 patients and portal hypertension w/o PAH 810 patients after propensity score matching. (a) The median time to hospitalization related to cardiovascular diseases was 54.1 months for PoPH group and could not be derived for portal hypertension w/o PAH group (*P* < 0.001 by log‐rank test). (d) The median time to hospitalization related to all cause was 8.0 months for PoPH group and 15.9 months for portal hypertension w/o PAH (*P* < 0.001 by log‐rank test). (e) The median time to all‐cause death was 55.4 months for PoPH group and 61.4 months for portal hypertension w/o PAH group.

The results of the six defined adverse events in PoPH and portal hypertension w/o PAH groups are shown in Figure 2, Tables S5 and S6, and Figures S1‐A–S1‐C, Supporting information. The median time to all‐cause hospitalization (Fig. 2d) was 8.0 months (95% CI: 6.0–13.7) for PoPH group, and 15.9 months (95% CI: 13.0–19.6) for portal hypertension w/o PAH (*P* < 0.001 by log‐rank test). In the median time to all‐cause hospitalization of Child–Pugh classification, there was a statistically significant difference between the two groups in Class A in Figure S1‐A(d), Supporting information (PoPH group, 18.2 months [95% CI: 7.2–43.9]; portal hypertension w/o PAH group, 32.7 months [95% CI: 22.6–47.9, *P* = 0.036 by log‐rank test]) and Class B in Figure S1‐B(d) (PoPH group, 6.5 months [95% CI: 4.3–11.9]; portal hypertension w/o PAH group; 13.0 months [95% CI: 10.3–17.0, *P* = 0.004 by log‐rank test]). For Child–Pugh Class C in Figure S1‐C(d), no meaningful difference was observed between the two groups.

### The potential cause of death

Among 125 PoPH patients with death outcome, congestive heart failure/other and unspecified liver cirrhosis were more frequent in eight patients (6.4%) followed by primary pulmonary hypertension in six patients (4.8%) (Table 2).

**Table 2:** Injuries and diseases in which the most medical resources were invested for the deceased cases

		PoPH (*N* = 386)
ICD‐10 code	ICD‐10	*n* (%)
Death		
	No	261 (67.6)
	Yes	125 (32.4)
I500	Congestive heart failure	8 (6.4)
K746	Other and unspecified cirrhosis of liver	8 (6.4)
I270	Primary pulmonary hypertension	6 (4.8)
B182	Chronic viral hepatitis C	3 (2.4)
C220	Malignant neoplasm: Liver cell carcinoma	3 (2.4)
I469	Cardiac arrest, unspecified	3 (2.4)
J841	Other interstitial pulmonary diseases with fibrosis	3 (2.4)
K767	Hepatorenal syndrome	2 (1.6)
A047, A419, A499	bacterial intestinal infections, sepsis, bacterial infection	1 each (0.8 each)
C021, C343, C541, C844, C900, C915	Malignant neoplasm	1 each (0.8 each)
D71	Functional disorders of polymorphonuclear neutrophils	1 (0.8)
E880	Disorders of plasma‐protein metabolism, not elsewhere classified	1 (0.8)
I050, I071, I330, I499, I501, I509, I615, I619, I850	Chronic rheumatic heart diseases, Other forms of heart disease, intracerebral hemorrhage, esophageal varices	1 each (0.8 each)
J159, J189, J431, J991	Bacterial pneumonia, pneumonia, emphysema, respiratory disorders in other diffuse connective tissue disorders	1 each (0.8 each)
K658, K703, K720, K721, K729, K761	Peritonitis, disease of the liver	1 each (0.8 each)
M329, M340, M348	Systemic lupus erythematosus, systemic sclerosis	1 each (0.8 each)
S3200	Fracture of lumbar vertebra	1 (0.8)

ICD‐10, international statistical classification of diseases and related health problems 10th revision; PoPH, portopulmonary hypertension.

### Treatment pattern

**Figure 3:**
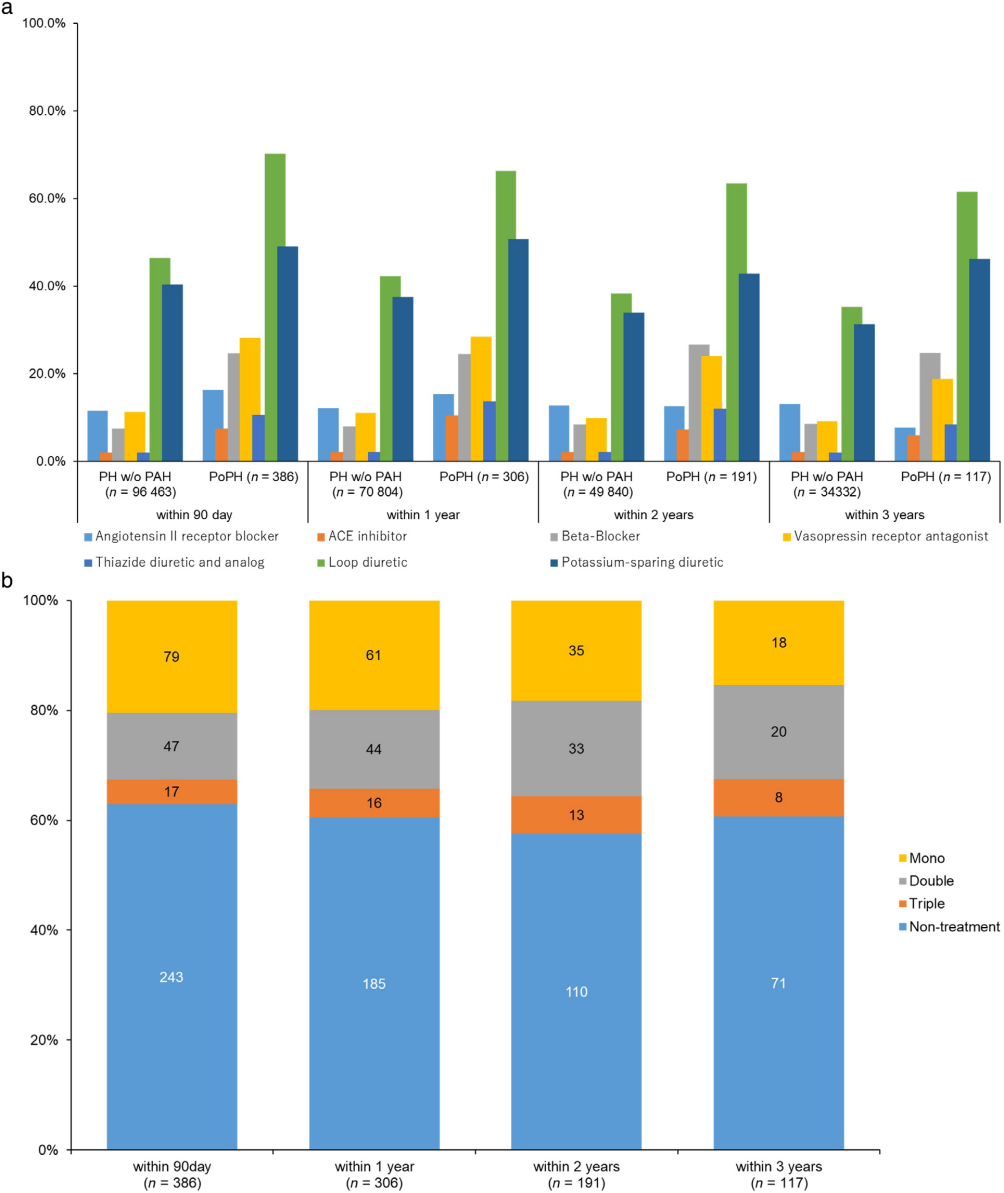
(a) Treatment pattern of portal hypertension medicine in portal hypertension and PoPH patients. In both portal hypertension w/o PAH and PoPH groups, the proportion of loop diuretics and potassium‐sparing diuretics was high, and the pattern was the same within 90 days to 3 years. However, PoPH group used more vasopressin receptor antagonist. (b) Treatment pattern of PAH‐specific medicine in PoPH patients. About 60% of PoPH patients did not receive PAH‐specific treatment throughout the entire observation period.

In PoPH group of 386 patients, loop diuretics had the highest treatment preference (271, 70.2%) within 90 days (Fig. 3a). The combined percentage of patients in PoPH group who received mono therapy or combination therapy of pulmonary vasodilators was 143 (37.0%) within 90 days, less than half of the total. Monotherapy (79, 20.5%) of prostacyclin (PGI2) (oral [po]/inhaled) (34, 8.8%) was most common in PoPH group (386 patients) within 90 days (Fig. 3b). In combination therapy (64, 16.6%), endothelin receptor antagonist (ERA) + nitric oxide (NO) (27, 7.0%) was the most frequent option within 90 days (Table S7, Supporting information).

### Other analysis

The most frequent complication was malignancy in both groups, but the incidence was higher in PoPH group than that of portal hypertension w/o PAH group except for malignancy (Table 3).

**Table 3:** Incidence rate of complications

	PH w/o PAH			PoPH		
	(*N* = 96 463)			(*N* = 386)		
Complications	person‐years	# of events	/1000 person‐years (95% CI)	person‐years	# of events	/1000 person‐years (95% CI)
Chronic pulmonary disease	150 057.1	17 607	117.3 (115.6–119.1)	392.0	144	367.4 (307.4–427.4)
Rheumatic disease	165 600.9	8580	51.8 (50.7–52.9)	404.0	127	314.4 (259.7–369.1)
Diabetes	135 834.9	25 897	190.7 (188.3–193.0)	490.7	113	230.3 (187.8–272.7)
Renal disease	165 007.4	12 376	75.0 (73.7–76.3)	477.8	111	232.3 (189.1–275.5)
Any malignancy	44 857.1	67 399	1502.5 (1491.2–1513.9)	240.1	243	1012.2 (884.9–1139.5)

CI, confidence interval; PH w/o PAH, portal hypertension without pulmonary arterial hypertension; PoPH, portopulmonary hypertension.

## Discussion

To the authors' knowledge, this is the first study to analyze the patient characteristics, treatment patterns, prognosis, and causes of death using large‐scale administrative claim database to clarify the characteristics of PoPH patients in Japan.

### Clinical characteristics of PoPH in Japan

The number of patients with PoPH in this study was 386 (0.4%, 386/96 849), a relative minor population. Considering that the proportion of PoPH patients in previous studies was 1.1–6.3%,^1^, ^3^ the diagnosis rate in real‐world setting was low in Japan. Although the results of this study cannot be directly compared with previous reports because of variation, including diverse life styles, inherent differences in genetic background, etc., among patients from different countries,^1^, ^11^ the analysis of 386 PoPH patients is still considered informative for epidemiological studies due to the rarity of the disease. The patient characteristics of PoPH in Japan suggest that 68.9% of cases occurred in the population of 61‐year‐old or older, and females accounted for more than half the PoPH population (59.6%). This was consistent with previous reports from the United States.^1^ Regarding the comorbidities in PoPH group, hepatopulmonary syndrome and splenomegaly had a higher rate than others. Even though hepatopulmonary syndrome and PoPH were considered as different diseases, there had been case reports in which hepatopulmonary syndrome preceded PoPH and the two diseases occurred consecutively.^16^ Regarding splenomegaly, although the mechanism was not clear, Kawut *et al*.^1^ reported that most patients had splenomegaly in both PoPH group and the control group with liver disease. The presence of splenomegaly meant that splenic venous blood flow was increased, which may result in the formation of a portal hypervascular shunt in many cases. Ohno *et al*.^17^ reported that of nine congenital portal venous shunts (CPSVS), six were PAH. PAH was an important complication of CPSVS and potentially present in patients with PAH of unknown etiology. The etiologies of liver cirrhosis for patients in both PoPH and portal hypertension w/o PAH groups were mainly viral and metabolic causes, and patients with PoPH had a higher rate of PBC (13.7%). As indicated in the previous studies,^1^, ^3^ the proportion of rheumatic diseases was high in the results of this study (Table 3) as well, suggesting that the association between PAH and autoimmune diseases may exist.

### Outcome of PoPH patients

Under Child–Pugh classification, PoPH group was significantly at higher risk of hospitalization related to cardiovascular disease than portal hypertension w/o PAH group in all classes (each *P* < 0.001 by log‐rank test) as well as all‐cause hospitalizations (emergency hospitalization was also included) in Child–Pugh Class A (*P* = 0.036 by log‐rank test) and B (*P* = 0.004 by log‐rank test). Similar patterns of PoPH group in hospitalization related to cardiovascular disease and PAH (Fig. 2a,f) suggested a possible correlation between two events might exist. These results were consistent with previous reports in the United States,^18^ indicating the pulmonary hypertension group had a higher risk of all‐cause hospitalization. A median comparison of survival‐time analysis by Child–Pugh class showed no difference between PoPH group and portal hypertension w/o PAH group. Previous study reported that Child–Pugh Classes B and C in PoPH patients were associated with reduced survival.^19^ Differences between the results of this study and previous study may result from a limitation of the database used.

For the definition of death in this study, we counted patients whose outcome was death as a mortality event. However, not all mortality events are captured, and only inpatient deaths are recorded in the database. In a previous report from Mayo Clinic,^20^ PoPH patients had significantly poor 5‐year survival rates without treatment for pulmonary hypertension. All in all, PoPH had a poor outcome when treatments were absent, and early diagnosis and treatment were crucial to patient's well‐being and survival.

Regarding PoPH patients with death outcome despite the input of huge amount of medical resources, the proportions of death resulting from cardiovascular and liver disease were nearly the same. In the report from the United States, a large proportion of deaths were attributed to liver disease, and PoPH might also be involved, suggesting that complications of PAH might have a role in patient's mortality.^21^

### Treatment patterns of diuretics and others

This study also revealed the treatment patterns for portal hypertension w/o PAH and PoPH groups in the real clinical setting. In PoPH group, looped diuretics were more often used within 90 days and within 1–3 years (Tables S7 and S8). Regarding the high percentage of the use of loop diuretics and potassium‐sparing diuretics in the present results, it is possible that the increased percentage of use was due to cardiovascular diseases caused by PoPH. Vasopressin receptor antagonist has been reported to have adequate efficacy and safety to address the fluid retention problem in cirrhosis,^22^ and low‐dose tolvaptan has been reported to be effective for PoPH patients associated with liver cirrhosis in Japan.^23^ However, due to the limited number of patients, more information was needed to draw a better conclusion. The trend of treatment pattern of combination therapy within 1–3 years in PoPH group was similar to the trend of within 90 days (Tables S7 and S8).

### Treatment pattern of pulmonary vasodilators for PoPH group

In treatment pattern analysis of pulmonary vasodilators, the proportion of patients who received combination therapy did not change much within 1–3 years compared with within 90 days (Tables S7–S9). It is also worth noting that the percentage of patients in PoPH group who did not receive any PAH‐specific medicine was 63.0% within 90 days (243 patients), which meant that more than 50% of the patients were untreated during this time span (Table S7). In this study, only 37.0% of the PoPH group had been prescribed PAH‐specific medicine at baseline, whereas a previous study^24^ reported that 84.6% had been prescribed PAH‐specific medicine. In this study, we collected a wide range of data from the administrative claim database, so it is assumed that there were differences between this study and the previous study in the proportion of patients receiving PAH treatment. Although PAH‐targeted therapies may help patients treat PoPH, the medications should be carefully considered.^25^ Because of potential issues over liver function in PoPH patients, careful initiation of mono therapy in the early phase was required.^12^ In monotherapy, Beraprost usage had the highest percentages within 90 days. Efficacy of Beraprost in PAH has also been reported in ALPHABET study, but its effect was still unclear on PoPH. However, the safety profile of the drug was excellent with no observed systemic hypotension and hepatic, renal, or hematologic side effects, which indicates that the drug may be safe for PoPH patients.^26^ Among ERA, Macitentan use was more common within 90 days. The data period of this study spanned the one prior to the approval date of Macitentan, so it was anticipated that the use of ERA would increase in the future.

In combination therapy, ERA+NO combination was the most prevalent with top choices of Macitentan+Tadalafil within 90 days. In Japan, there was a case report claiming that the combination of Ambisentan+Tadalafil was effective for PoPH, but more information was needed due to the small sample size.^27^ In a recent Japanese registry study, more than 60% of PoPH patients (22 patients) were prescribed combination therapy, with ERA+NO being the most common (36.4%).^28^

### Incidence of complications

The incidence of complications, except for malignancy, tended to be higher in PoPH group than portal hypertension w/o PAH group. There were previous reports on complications of PoPH compared with other liver diseases and idiopathic/heritable‐PAH, but no significant difference was found between the two groups.^12^ Although there were many reports on the etiology of cirrhosis in PoPH,^1^, ^2^, ^3^, ^5^, ^7^, ^18^, ^20^, ^21^ few reports on the presence or absence of comorbidities and the incidence of complications existed. This study manifested that the prevalence of the complications was higher in PoPH group than portal hypertension w/o PAH group, including chronic pulmonary disease, rheumatic disease, diabetes, and renal disease (Table 3).

### Limitations of this study

This study has the following limitations. (i) Absence of valid studies precluded the attestation of the definition of the study population and precision of the outcome. (ii) It was impossible to match the severity of pulmonary hypertension due to the small number of labo data and NYHA classification data. (iii) It was impossible to learn the history of medical treatment and prescription before the medical examination if prior medical care information and medical examination were from the hospitals outside the DPC system. (iv) If the diagnosis name was entered for the purpose of insurance claims, it might differ from the actual diagnosis name. (v) Since the Index date was not always the diagnosis date, the first day overlapping between the definitive diagnosis records of PAH, echocardiography, or right cardiac catheterization was assumed to be the diagnosis date. (vi) Information about deaths outside hospitals was not collected.

## Conclusion

This is the first study to characterize PoPH patient characteristics, treatment patterns, prognosis, and hospitalization period by disease factor in Japan. Patients with PoPH were generally older and more likely to be female. The etiology of cirrhosis was characterized as viral cause being the most common and the proportion of PBC being high in PoPH patients compared with that in patients with portal hypertension w/o PAH. Treatment patterns showed a high proportional use of diuretic and vasopressin receptor antagonist, but the percentage of pulmonary vasodilator use among patients with PoPH was low. Since PoPH is a rare disease, information on epidemiology and treatment method was insufficient. The findings of this study may improve PoPH disease awareness and medical care in the future.

## Supporting information


**Appendix S1.** Supporting Information.
**Table S1.** Disease name.
**Table S2.** Medical procedure name.
**Table S3.** Treatment patterns.

**Table S4.** Patient characteristics on laboratory data.
**Table S5.** Time to event survival analysis.
**Table S6‐A.** Time to event survival analysis (Child‐Pugh class A).
**Table S6‐B.** Time to event survival analysis (Child‐Pugh class B).
**Table S6‐C.** Time to event survival analysis (Child‐Pugh class C).
**Table S7.** Liver disease and pulmonary vasodilator treatment pattern (PH w/o PAH *versus* PoPH).
**Table S8.** Liver disease and pulmonary vasodilator treatment pattern by matched cohort (PH w/o PAH *versus* PoPH).
**Table S9.** Treatment pattern subdivided into generic names of pulmonary vasodilator.
**Figure S1‐A.** Time to event survival analysis (Kaplan Meier plot) (Child‐Pugh class A). (a) The time to emergency hospitalization related to cardiovascular disease was significantly different between PoPH group and portal hypertension w/o PAH group (*P* < 0.001 by log‐rank test). (d) The median of time to all cause emergency hospitalization was 18.2 months for PoPH group and 32.7 months for portal hypertension w/o PAH group (*P* = 0.036 by log‐rank test).
**Figure S1‐B.** Time to event survival analysis (Kaplan Meier plot) (Child‐Pugh class B). (a) The median of time to emergency hospitalization attributable to cardiovascular diseases was 54.1 months for PoPH group (*P* < 0.001 by log‐rank test). (d) The median of time to all cause emergency hospitalization was 6.5 months for PoPH group and 13.0 months for portal hypertension w/o PAH group (*P* = 0.004 by log‐rank test).
**Figure S1‐C.** Time to event survival analysis (Kaplan Meier plot) (Child‐Pugh class C). (a) The median of time to emergency hospitalization attributable to cardiovascular diseases was 30.8 months for PoPH group and could not be derived for portal hypertension w/o PAH group (*P* < 0.001 by log‐rank test).

## Data Availability

The datasets used in this study were purchased from Medical Data Vision Co., Ltd. The study data are available on request from the authors.
