# Supplementary material for: Treatment pattern and clinical outcomes in portopulmonary hypertension: A database study in Japan
Source: JGH Open. 2022 Oct 13;6(11):763–73. doi: 10.1002/jgh3.12820 (PMC9667400; doi:10.1002/jgh3.12820)
Supplement: Supplementary file 2 — Table S4. Patient characteristics on laboratory data. Table S5. Time to event survival analysis. Table S6‐A. Time to event survival analysis (Child‐Pugh class A). Table S6‐B. Time to event survival analysis (Child‐Pugh class B). Table S6‐C. Time to event survival analysis (Child‐Pugh class C). Table S7. Liver disease and pulmonary vasodilator treatment pattern (PH w/o PAH versus PoPH). Table S8. Liver disease and pulmonary vasodilator treatment pattern by matched cohort (PH w/o PAH versus PoPH). Table S9. Treatment pattern subdivided into generic names of pulmonary vasodilator. Figure S1‐A. Time to event survival analysis (Kaplan Meier plot) (Child‐Pugh class A). (a) The time to emergency hospitalization related to cardiovascular disease was significantly different between PoPH group and portal hypertension w/o PAH group (P < 0.001 by log‐rank test). (d) The median of time to all cause emergency hospitalization was 18.2 months for PoPH group and 32.7 months for portal hypertension w/o PAH group (P = 0.036 by log‐rank test). Figure S1‐B. Time to event survival analysis (Kaplan Meier plot) (Child‐Pugh class B). (a) The median of time to emergency hospitalization attributable to cardiovascular diseases was 54.1 months for PoPH group (P < 0.001 by log‐rank test). (d) The median of time to all cause emergency hospitalization was 6.5 months for PoPH group and 13.0 months for portal hypertension w/o PAH group (P = 0.004 by log‐rank test). Figure S1‐C. Time to event survival analysis (Kaplan Meier plot) (Child‐Pugh class C). (a) The median of time to emergency hospitalization attributable to cardiovascular diseases was 30.8 months for PoPH group and could not be derived for portal hypertension w/o PAH group (P < 0.001 by log‐rank test). [file JGH3-6-763-s002.docx]

**Treatment pattern and clinical outcomes in portopulmonary hypertension: a database study in Japan**

**Journal: JGH Open**

**Supporting information - Tables & Figures**

Masanori Atsukawa^*^, Masashi Takano^†^, Junichi Omura^†^

^*^ Division of Gastroenterology and Hepatology, Nippon Medical School, 1-1-5, Sendagi, Bunkyo-ku, Tokyo, 113-8603, Japan.

Phone: +81-3-3822-2131

E-mail: [momogachi@yahoo.co.jp](mailto:momogachi@yahoo.co.jp)

^†^ Janssen Pharmaceutical K.K. Medical affairs division. 3-5-2 Nishikanda, Chiyoda-ku, Tokyo 101-0065, Japan.

Phone: +81-80-7839-2970

Masashi Takano

E-mail: [mtakano4@ITS.JNJ.com](file:///\\acrosv01\ヤンセン_DB研究_PAH\03_MW\06_作成\03.review\社内レビュー／QC\20211014_最終版\対象文書\5次QC\mtakano4@ITS.JNJ.com)

Junichi Omura

E-mail: [JOmura@ITS.JNJ.com](mailto:JOmura@ITS.JNJ.com)

**Supporting information Tables & Figures**

- **Table S4** Patient characteristics on laboratory data
- **Table S5** Time to event survival analysis
- **Table S6-A** Time to event survival analysis (Child-Pugh class A)
- **Table S6-B** Time to event survival analysis (Child-Pugh class B)
- **Table S6-C** Time to event survival analysis (Child-Pugh class C)
- **Table S7** Liver disease and pulmonary vasodilator treatment pattern (PH w/o PAH versus PoPH)
- **Table S8** Liver disease and pulmonary vasodilator treatment pattern by matched cohort (PH w/o PAH versus PoPH)
- **Table S9** Treatment pattern subdivided into generic names of pulmonary vasodilator
- **Figure S1-A** Time to event survival analysis (Kaplan Meier plot) (Child-Pugh class A)
- **Figure S1-B** Time to event survival analysis (Kaplan Meier plot) (Child-Pugh class B)
- **Figure S1-C** Time to event survival analysis (Kaplan Meier plot) (Child-Pugh class C)

Details of (a) to (f) of Figure S1-A to S1-C are as follows (emergency hospitalization was referred to as hospitalization).

- **(a)** Time to hospitalization related to cardiovascular disease survival analysis
- **(b)** Time to hospitalization related to liver disease survival analysis
- **(c)** Time to hospitalization related to renal failure survival analysis
- **(d)** Time to all cause hospitalization survival analysis
- **(e)** Time to all cause death survival analysis
- **(f)** Time to hospitalization related to PAH survival analysis

**Table S4. Patient characteristics on laboratory data**

|  |  | **PH w/o PAH** | | **PoPH** | | **PH w/o PAH (matched cohort)** | | **PoPH (matched cohort)** | |
| --- | --- | --- | --- | --- | --- | --- | --- | --- | --- |
|  |  | **n** | **(%)** | **n** | **(%)** | **n** | **(%)** | **n** | **(%)** |
| Total |  | 96463 | | 386 | | 840 | | 210 | |
| Labo Data | |  |  |  |  |  |  |  |  |
| BNP [pg/mL] | |  |  |  |  |  |  |  |  |
|  | n | 899 | | 14 | | 7 | | 6 | |
|  | mean (S.D.) | 252.57 (636.11) | | 322.27 (259.15) | | 120.16 (109.92) | | 460.35 (311.43) | |
|  | min - med -max | 3.8 - 91.70 - 9553.0 | | 44.3 - 248.00 - 833.4 | | 7.8 - 99.60 - 337.9 | | 104.5 - 489.20 - 833.4 | |
| NT-proBNP [pg/mL] | |  |  |  |  |  |  |  |  |
|  | n | 284 | | 5 | | 2 | | 3 | |
|  | mean (S.D.) | 1830.64 (6547.50) | | 6962.80 (12719.37) | | 2372.00 (2901.97) | | 958.33 (845.53) | |
|  | min - med -max | 14.0 - 334.00 - 99130.0 | | 96.0 - 1786.00 - 29668.0 | | 320.0 - 2372.00 - 4424.0 | | 96.0 - 993.00 - 1786.0 | |
| Hemoglobin [g/dL] | |  |  |  |  |  |  |  |  |
|  | n | 7970 | | 30 | | 62 | | 15 | |
|  | mean (S.D.) | 11.22 (2.27) | | 10.73 (1.91) | | 10.85 (1.91) | | 11.06 (1.58) | |
|  | min - med -max | 2.8 - 11.20 - 19.9 | | 6.7 - 11.05 - 13.5 | | 7.2 - 10.90 - 14.9 | | 8.7 - 11.10 - 13.5 | |
| Albumin (serum) [g/dL] | |  |  |  |  |  |  |  |  |
|  | n | 7781 | | 30 | | 61 | | 15 | |
|  | mean (S.D.) | 3.02 (0.75) | | 3.23 (0.72) | | 2.97 (0.68) | | 3.10 (0.73) | |
|  | min - med -max | 0.3 - 3.00 - 5.4 | | 1.7 - 3.25 - 4.8 | | 1.4 - 2.90 - 4.5 | | 1.7 - 3.10 - 4.5 | |
| Creatinine (serum) [mg/dL] | |  |  |  |  |  |  |  |  |
|  | n | 7930 | | 30 | | 62 | | 15 | |
|  | mean (S.D.) | 1.08 (1.26) | | 1.16 (0.87) | | 1.21 (1.66) | | 0.96 (0.60) | |
|  | min - med -max | 0.1 - 0.77 - 16.0 | | 0.5 - 0.89 - 4.5 | | 0.3 - 0.78 - 12.4 | | 0.5 - 0.86 - 3.0 | |
| Uric acid (serum) [mg/dL] | |  |  |  |  |  |  |  |  |
|  | n | 5613 | | 29 | | 46 | | 15 | |
|  | mean (S.D.) | 5.80 (2.28) | | 6.74 (2.29) | | 5.85 (2.37) | | 6.80 (1.61) | |
|  | min - med -max | 0.4 - 5.60 - 23.2 | | 2.4 - 6.60 - 12.8 | | 0.6 - 5.65 - 14.8 | | 3.9 - 6.60 - 10.9 | |
| Total bilirubin (serum) [g/dL] | |  |  |  |  |  |  |  |  |
|  | n | 7864 | | 30 | | 61 | | 15 | |
|  | mean (S.D.) | 2.01 (3.01) | | 1.36 (1.25) | | 1.53 (1.33) | | 1.15 (0.99) | |
|  | min - med -max | 0.1 - 1.14 - 34.5 | | 0.2 - 0.90 - 5.6 | | 0.3 - 1.20 - 6.7 | | 0.3 - 0.90 - 3.5 | |
| Platelets [x10000/μL] | |  |  |  |  |  |  |  |  |
|  | n | 7970 | | 30 | | 62 | | 15 | |
|  | mean (S.D.) | 13.49 (8.75) | | 9.29 (3.92) | | 11.48 (5.01) | | 9.03 (2.85) | |
|  | min - med -max | 0.5 - 11.20 - 140.2 | | 0.8 - 9.05 - 16.7 | | 1.4 - 10.60 - 24.3 | | 4.0 - 8.90 - 15.8 | |
| Prothrombin time [%] | |  |  |  |  |  |  |  |  |
|  | n | 5351 | | 17 | | 40 | | 10 | |
|  | mean (S.D.) | 70.56 (20.02) | | 60.38 (18.11) | | 68.29 (15.61) | | 58.47 (11.26) | |
|  | min - med -max | 3.0 - 71.00 - 163.8 | | 26.0 - 61.00 - 100.0 | | 32.0 - 69.15 - 100.0 | | 35.2 - 59.45 - 75.0 | |
| Abbreviations: PH, portal hypertension; PoPH, portopulmonary hypertension; PH w/o PAH, portal hypertension without pulmonary arterial hypertension; PoPH, portopulmonary hypertension; BNP, brain natriuretic peptide; NT-proBNP, N-terminal pro-brain natriuretic peptide; S.D., standard deviation. | | | | | | | | | |

**Table S5. Time to event survival analysis**

|  |  | **PH w/o PAH (matched cohort)** | | **PoPH (matched cohort)** | |
| --- | --- | --- | --- | --- | --- |
|  |  | **(N = 840)** | | **(N = 210)** | |
| 1)Time to hospitalization† related to cardiovascular disease [days] | |  |  |  |  |
|  | n | 31 | | 60 | |
|  | mean (S.D.) | 623.8 (701.9) | | 319.1 (432.4) | |
|  | min - med - max | 61 - 474.0 - 3610 | | 3 - 116.5 - 1918 | |
| 2) Time to hospitalization† related to liver disease [days] | |  |  |  |  |
|  | n | 278 | | 57 | |
|  | mean (S.D.) | 483.3 (564.2) | | 371.1 (422.5) | |
|  | min - med - max | 29 - 271.5 - 3045 | | 5 - 224.0 - 1722 | |
| 3) Time to hospitalization† related to renal failure [days] | |  |  |  |  |
|  | n | 14 | | 5 | |
|  | mean (S.D.) | 379.6 (438.9) | | 790.8 (730.0) | |
|  | min - med - max | 29 - 234.5 - 1717 | | 143 - 398.0 - 1610 | |
| 4) Time to all cause hospitalization† [days] | |  |  |  |  |
|  | n | 455 | | 130 | |
|  | mean (S.D.) | 450.9 (508.7) | | 280.3 (385.5) | |
|  | min - med - max | 29 - 268.0 - 2890 | | 3 - 125.0 - 1918 | |
| 5) Time to all cause death [days] | |  |  |  |  |
|  | n | 273 | | 72 | |
|  | mean (S.D.) | 562.8 (622.3) | | 395.7 (446.6) | |
|  | min - med - max | 1 - 320.0 - 2620 | | 8 - 223.5 - 1865 | |
| 6) Time to hospitalization† related to PAH [days] | |  |  |  |  |
|  | n |  | | 47 | |
|  | mean (S.D.) |  | | 280.0 (433.0) | |
|  | min - med - max |  | | 3 - 93.0 - 1918 | |
| Abbreviations: PH w/o PAH, portal hypertension without pulmonary arterial hypertension; PoPH, portopulmonary hypertension: PAH, pulmonary arterial hypertension; S.D., standard deviation; min - med - max, minimum - median - maximum..  †: Emergency hospitalization was referred to as hospitalization. | | | | | |

**Table S6-A. Time to event survival analysis (Chiid-Pugh class A)**

| **Child–Pugh class A** | | **PH w/o PAH (matched cohort)** | | **PoPH (matched cohort)** | |
| --- | --- | --- | --- | --- | --- |
|  |  | **(N=221)** | | **(N=59)** | |
| 1) Time to hospitalization† related to cardiovascular disease [days] | |  |  |  |  |
|  | n | 8 | | 16 | |
|  | mean (S.D.) | 1048.4 (1163.1) | | 432.0 (466.5) | |
|  | min - med -max | 65 - 764.5 - 3610 | | 22 - 175.0 - 1375 | |
| 2) Time to hospitalization† related to liver disease [days] | |  |  |  |  |
|  | n | 53 | | 9 | |
|  | mean (S.D.) | 634.0 (718.7) | | 670.0 (648.0) | |
|  | min - med -max | 46 - 382.0 - 3045 | | 23 - 513.0 - 1722 | |
| 3) Time to hospitalization† related to renal failure [days] | |  |  |  |  |
|  | n | 3 | | 2 | |
|  | mean (S.D.) | 892.3 (746.4) | | 927.5 (965.2) | |
|  | min - med -max | 263 - 697.0 - 1717 | | 245 - 927.5 - 1610 | |
| 4) Time to all cause hospitalization† [days] | |  |  |  |  |
|  | n | 96 | | 33 | |
|  | mean (S.D.) | 563.4 (584.9) | | 380.9 (465.4) | |
|  | min - med -max | 31 - 353.0 - 2890 | | 22 - 191.0 - 1722 | |
| 5) Time to all cause death [days] | |  |  |  |  |
|  | n | 28 | | 13 | |
|  | mean (S.D.) | 583.0 (646.0) | | 723.6 (557.6) | |
|  | min - med -max | 1 - 408.5 - 2252 | | 227 - 482.0 - 1865 | |
| 6) Time to hospitalization† related to PAH [days] | |  |  |  |  |
|  | n |  |  | 11 | |
|  | mean (S.D.) |  |  | 371.6 (457.2) | |
|  | min - med -max |  |  | 22 - 93.0 - 1335 | |
| Abbreviations: PH w/o PAH, portal hypertension without pulmonary arterial hypertension; PoPH, portopulmonary hypertension; PAH, pulmonary arterial hypertension; S.D., standard deviation; min, minimum; med, median; max, maximum.  †: Emergency hospitalization was referred to as hospitalization. | | | | | |

**Table S6-B. Time to event survival analysis (Child-Pugh class B)**

| **Child–Pugh class B** | | **PH w/o PAH (matched cohort)** | | **PoPH (matched cohort)** | |
| --- | --- | --- | --- | --- | --- |
|  |  | **(N=446)** | | **(N=109)** | |
| 1) Time to hospitalization† related to cardiovascular disease [days] | |  |  |  |  |
|  | n | 16 | | 32 | |
|  | mean (S.D.) | 526.4 (403.3) | | 283.6 (451.7) | |
|  | min - med -max | 153 - 418.5 - 1440 | | 3 - 98.0 - 1918 | |
| 2) Time to hospitalization† related to liver disease [days] | |  |  |  |  |
|  | n | 170 | | 30 | |
|  | mean (S.D.) | 453.1 (505.1) | | 319.9 (340.3) | |
|  | min - med -max | 29 - 267.5 - 2559 | | 7 - 227.0 - 1405 | |
| 3) Time to hospitalization† related to renal failure [days] | |  |  |  |  |
|  | n | 9 | | 3 | |
|  | mean (S.D.) | 263.2 (207.1) | | 699.7 (754.2) | |
|  | min - med -max | 29 - 205.0 - 645 | | 143 - 398.0 - 1558 | |
| 4) Time to all cause hospitalization† [days] | |  |  |  |  |
|  | n | 274 | | 71 | |
|  | mean (S.D.) | 420.2 (462.6) | | 236.0 (351.5) | |
|  | min - med -max | 29 - 254.5 - 2559 | | 3 - 102.0 - 1918 | |
| 5) Time to all cause death [days] | |  |  |  |  |
|  | n | 153 | | 40 | |
|  | mean (S.D.) | 687.1 (645.9) | | 329.2 (398.8) | |
|  | min - med -max | 1 - 481.0 - 2620 | | 9 - 183.5 - 1685 | |
| 6) Time to hospitalization† related to PAH [days] | |  |  |  |  |
|  | n |  |  | 26 | |
|  | mean (S.D.) |  |  | 274.3 (479.6) | |
|  | min - med -max |  |  | 3 - 92.0 - 1918 | |
| Abbreviations: PH w/o PAH, portal hypertension without pulmonary arterial hypertension; PoPH, portopulmonary hypertension; PAH, pulmonary arterial hypertension; S.D., standard deviation; min, minimum; med, median; max, maximum.  †: Emergency hospitalization was referred to as hospitalization. | | | | | |

**Table S6-C. Time to event survival analysis (Child-Pugh class C)**

| **Child–Pugh class C** | | **PH w/o PAH (matched cohort)** | | **PoPH (matched cohort)** | |
| --- | --- | --- | --- | --- | --- |
|  |  | **(N=173)** | | **(N=42)** | |
| 1) Time to hospitalization† related to cardiovascular disease [days] | |  |  |  |  |
|  | n | 7 | | 12 | |
|  | mean (S.D.) | 361.1 (364.0) | | 263.1 (324.7) | |
|  | min - med -max | 61 - 148.0 - 1043 | | 5 - 125.0 - 938 | |
| 2) Time to hospitalization† related to liver disease [days] | |  |  |  |  |
|  | n | 55 | | 18 | |
|  | mean (S.D.) | 431.4 (556.1) | | 306.9 (369.4) | |
|  | min - med -max | 34 - 215.0 - 2599 | | 5 - 129.5 - 1191 | |
| 3) Time to hospitalization† related to renal failure [days] | |  |  |  |  |
|  | n | 2 | | 0 | |
|  | mean (S.D.) | 134.0 (124.5) | | - | |
|  | min - med -max | 46 - 134.0 - 222 | | - | |
| 4) Time to all cause hospitalization† [days] | |  |  |  |  |
|  | n | 85 | | 26 | |
|  | mean (S.D.) | 422.5 (545.4) | | 273.7 (353.2) | |
|  | min - med -max | 31 - 241.0 - 2729 | | 5 - 112.5 - 1191 | |
| 5) Time to all cause death [days] | |  |  |  |  |
|  | n | 92 | | 19 | |
|  | mean (S.D.) | 350.1 (514.8) | | 311.3 (373.8) | |
|  | min - med -max | 1 - 96.0 - 2460 | | 8 - 179.0 - 1219 | |
| 6) Time to hospitalization† related to PAH [days] | |  |  |  |  |
|  | n |  |  | 10 | |
|  | mean (S.D.) |  |  | 193.9 (261.9) | |
|  | min - med -max |  |  | 5 - 92.5 - 757 | |
| Abbreviations: PH w/o PAH, portal hypertension without pulmonary arterial hypertension; PoPH, portopulmonary hypertension; PAH, pulmonary arterial hypertension; S.D., standard deviation; min, minimum; med, median; max, maximum.  †: Emergency hospitalization was referred to as hospitalization. | | | | | |

**Table S7. Liver disease and pulmonary vasodilator treatment pattern (PH w/o PAH versus PoPH)**

|  |  |  | within 90 days | | | | within 1 year | | | | within 2 years | | | | within 3 years | | | |
| --- | --- | --- | --- | --- | --- | --- | --- | --- | --- | --- | --- | --- | --- | --- | --- | --- | --- | --- |
|  |  |  | PH w/o PAH | | PoPH | | PH w/o PAH | | PoPH | | PH w/o PAH | | PoPH | | PH w/o PAH | | PoPH | |
| Treatment Pattern | | | (N=96463) | | (N=386) | | (N=70804) | | (N=306) | | (N=49840) | | (N=191) | | (N=34332) | | (N=117) | |
|  |  |  | n | (%) | n | (%) | n | (%) | n | (%) | n | (%) | n | (%) | n | (%) | n | (%) |
| PAH Specific Medicine | | |  |  |  |  |  |  |  |  |  |  |  |  |  |  |  |  |
|  |  | Mono therapy (all) |  |  |  |  |  |  |  |  |  |  |  |  |  |  |  |  |
|  |  | ERA |  |  | 24 | (6.2) |  |  | 17 | (5.6) |  |  | 17 | (8.9) |  |  | 8 | (6.8) |
|  |  | NO |  |  | 20 | (5.2) |  |  | 17 | (5.6) |  |  | 8 | (4.2) |  |  | 5 | (4.3) |
|  |  | PGI2(po/inhaled) |  |  | 34 | (8.8) |  |  | 27 | (8.8) |  |  | 10 | (5.2) |  |  | 5 | (4.3) |
|  |  | PGI2(sc/iv) |  |  | 1 | (0.3) |  |  | 0 | (0.0) |  |  | 0 | (0.0) |  |  | 0 | (0.0) |
|  |  |  |  |  |  |  |  |  |  |  |  |  |  |  |  |  |  |  |
|  |  | Combination therapy (all) |  |  |  |  |  |  |  |  |  |  |  |  |  |  |  |  |
|  |  | ERA + NO |  |  | 27 | (7.0) |  |  | 28 | (9.2) |  |  | 20 | (10.5) |  |  | 14 | (12.0) |
|  |  | ERA + PGI2(po/inhaled) |  |  | 7 | (1.8) |  |  | 6 | (2.0) |  |  | 5 | (2.6) |  |  | 3 | (2.6) |
|  |  | ERA + PGI2(sc/iv) |  |  | 0 | (0.0) |  |  | 0 | (0.0) |  |  | 0 | (0.0) |  |  | 0 | (0.0) |
|  |  | NO + PGI2(po/inhaled) |  |  | 13 | (3.4) |  |  | 9 | (2.9) |  |  | 8 | (4.2) |  |  | 3 | (2.6) |
|  |  | NO + PGI2(sc/iv) |  |  | 0 | (0.0) |  |  | 1 | (0.3) |  |  | 0 | (0.0) |  |  | 0 | (0.0) |
|  |  | PGI2(po/inhaled) + PGI2(sc/iv) |  |  | 0 | (0.0) |  |  | 0 | (0.0) |  |  | 0 | (0.0) |  |  | 0 | (0.0) |
|  |  | Triple |  |  | 17 | (4.4) |  |  | 16 | (5.2) |  |  | 13 | (6.8) |  |  | 8 | (6.8) |
| potassium-sparing diuretic | | | 38992 | (40.4) | 189 | (49.0) | 26519 | (37.5) | 155 | (50.7) | 16907 | (33.9) | 82 | (42.9) | 10732 | (31.3) | 54 | (46.2) |
| loop diuretic | | | 44755 | (46.4) | 271 | (70.2) | 29923 | (42.3) | 203 | (66.3) | 19109 | (38.3) | 121 | (63.4) | 12119 | (35.3) | 72 | (61.5) |
| thiazide diuretic and analog | | | 1968 | (2.0) | 41 | (10.6) | 1584 | (2.2) | 42 | (13.7) | 1109 | (2.2) | 23 | (12.0) | 695 | (2.0) | 10 | (8.5) |
| vasopressin receptor antagonist | | | 10884 | (11.3) | 109 | (28.2) | 7861 | (11.1) | 87 | (28.4) | 4953 | (9.9) | 46 | (24.1) | 3156 | (9.2) | 22 | (18.8) |
| Beta-Blocker | | | 7268 | (7.5) | 95 | (24.6) | 5634 | (8.0) | 75 | (24.5) | 4214 | (8.5) | 51 | (26.7) | 2964 | (8.6) | 29 | (24.8) |
| ACE inhibitor | | | 1944 | (2.0) | 29 | (7.5) | 1508 | (2.1) | 32 | (10.5) | 1086 | (2.2) | 14 | (7.3) | 748 | (2.2) | 7 | (6.0) |
| angiotensin II receptor blocker | | | 11121 | (11.5) | 63 | (16.3) | 8570 | (12.1) | 47 | (15.4) | 6329 | (12.7) | 24 | (12.6) | 4484 | (13.1) | 9 | (7.7) |
| Abbreviations: PH w/o PAH, portal hypertension without pulmonary arterial hypertension; PoPH, portopulmonary hypertension; ERA, endothelin receptor antagonist; NO,nitric oxide; PGI2(po/inhaled), prostacyclin (oral/inhaled); PGI2 (sc/iv), prostacyclin (subcutaneous/intravenous); ACE inhibitor, angiotensin converting enzyme inhibitor. | | | | | | | | | | | | | | | | | | |

**Table S8. Liver disease and pulmonary vasodilator treatment pattern by matched cohort (PH w/o PAH versus PoPH)**

|  |  |  | **within 90 days** | | | | **within 1 year** | | | | **within 2 years** | | | | **within 3 years** | | | |
| --- | --- | --- | --- | --- | --- | --- | --- | --- | --- | --- | --- | --- | --- | --- | --- | --- | --- | --- |
|  |  |  | PH w/o PAH (matched cohort) | | PoPH  (matched cohort) | | PH w/o PAH (matched cohort) | | PoPH  (matched cohort) | | PH w/o PAH (matched cohort) | | PoPH  (matched cohort) | | PH w/o PAH (matched cohort) | | PoPH  (matched cohort) | |
| Treatment Pattern | | | (N=840) | | (N=210) | | (N=650) | | (N=169) | | (N=475) | | (N=106) | | (N=327) | | (N=68) | |
|  |  |  | n | (%) | n | (%) | n | (%) | n | (%) | n | (%) | n | (%) | n | (%) | n | (%) |
| PAH Specific Medicine | | |  |  |  |  |  |  |  |  |  |  |  |  |  |  |  |  |
|  | Mono therapy (all) | |  |  |  |  |  |  |  |  |  |  |  |  |  |  |  |  |
|  |  | ERA |  |  | 13 | (6.2) |  |  | 9 | (5.3) |  |  | 9 | (8.5) |  |  | 6 | (8.8) |
|  |  | NO |  |  | 8 | (3.8) |  |  | 9 | (5.3) |  |  | 5 | (4.7) |  |  | 4 | (5.9) |
|  |  | PGI2 (po/inhaled) |  |  | 18 | (8.6) |  |  | 12 | (7.1) |  |  | 4 | (3.8) |  |  | 1 | (1.5) |
|  |  | PGI2 (sc/iv) |  |  | 0 | (0.0) |  |  | 0 | (0.0) |  |  | 0 | (0.0) |  |  | 0 | (0.0) |
|  |  |  |  |  |  |  |  |  |  |  |  |  |  |  |  |  |  |  |
|  | Combination therapy (all) | |  |  |  |  |  |  |  |  |  |  |  |  |  |  |  |  |
|  |  | ERA + NO |  |  | 19 | (9.0) |  |  | 19 | (11.2) |  |  | 11 | (10.4) |  |  | 10 | (14.7) |
|  |  | ERA + PGI2 (po/inhaled) |  |  | 3 | (1.4) |  |  | 2 | (1.2) |  |  | 2 | (1.9) |  |  | 1 | (1.5) |
|  |  | ERA + PGI2 (sc/iv) |  |  | 0 | (0.0) |  |  | 0 | (0.0) |  |  | 0 | (0.0) |  |  | 0 | (0.0) |
|  |  | NO + PGI2 (po/inhaled) |  |  | 11 | (5.2) |  |  | 8 | (4.7) |  |  | 8 | (7.5) |  |  | 3 | (4.4) |
|  |  | NO + PGI2 (sc/iv) |  |  | 0 | (0.0) |  |  | 1 | (0.6) |  |  | 0 | (0.0) |  |  | 0 | (0.0) |
|  |  | PGI2(po/inhaled) + PGI2 (sc/iv) |  |  | 0 | (0.0) |  |  | 0 | (0.0) |  |  | 0 | (0.0) |  |  | 0 | (0.0) |
|  | Triple | |  |  | 10 | (4.8) |  |  | 10 | (5.9) |  |  | 9 | (8.5) |  |  | 4 | (5.9) |
| potassium-sparing diuretic | | | 384 | (45.7) | 113 | (53.8) | 289 | (44.5) | 103 | (60.9) | 201 | (42.3) | 58 | (54.7) | 138 | (42.2) | 36 | (52.9) |
| loop diuretic | | | 422 | (50.2) | 155 | (73.8) | 314 | (48.3) | 119 | (70.4) | 203 | (42.7) | 77 | (72.6) | 145 | (44.3) | 47 | (69.1) |
| thiazide diuretic and analog | | | 19 | (2.3) | 22 | (10.5) | 16 | (2.5) | 25 | (14.8) | 7 | (1.5) | 14 | (13.2) | 8 | (2.4) | 7 | (10.3) |
| vasopressin receptor antagonist | | | 97 | (11.5) | 58 | (27.6) | 92 | (14.2) | 51 | (30.2) | 59 | (12.4) | 31 | (29.2) | 41 | (12.5) | 16 | (23.5) |
| Beta-Blocker | | | 72 | (8.6) | 43 | (20.5) | 64 | (9.8) | 34 | (20.1) | 44 | (9.3) | 26 | (24.5) | 32 | (9.8) | 17 | (25.0) |
| ACE inhibitor | | | 12 | (1.4) | 15 | (7.1) | 9 | (1.4) | 16 | (9.5) | 5 | (1.1) | 6 | (5.7) | 4 | (1.2) | 3 | (4.4) |
| angiotensin II receptor blocker | | | 121 | (14.4) | 33 | (15.7) | 85 | (13.1) | 25 | (14.8) | 60 | (12.6) | 11 | (10.4) | 42 | (12.8) | 4 | (5.9) |
| Abbreviations: PH w/o PAH, portal hypertension without pulmonary arterial hypertension; PoPH, portopulmonary hypertension; ERA, endothelin receptor antagonist; NO, nitric oxide; PGI2 (po/inhaled), prostacyclin (oral/inhaled); PGI2 (sc/iv), prostacyclin (subcutaneous/intravenous); ACE inhibitor, angiotensin converting enzyme inhibitor. | | | | | | | | | | | | | | | | | | |

**Table S9. Treatment pattern subdivided into generic names of pulmonary vasodilator**

|  |  | **within 90 days** | | **within 1 year** | | **within 2 years** | | **within 3 years** | |
| --- | --- | --- | --- | --- | --- | --- | --- | --- | --- |
| **Treatment Pattern (Generic name) PAH Specific Medicine** | | **PoPH** | | **PoPH** | | **PoPH** | | **PoPH** | |
|  |  | **(N=386)** | | **(N=306)** | | **(N=191)** | | **(N=117)** | |
|  |  | **n** | **(%)** | **n** | **(%)** | **n** | **(%)** | **n** | **(%)** |
| Mono therapy | |  |  |  |  |  |  |  |  |
| ERA | |  |  |  |  |  |  |  |  |
|  | Macitentan | 10 | (2.6) | 6 | (2.0) | 6 | (3.1) | 2 | (1.7) |
|  | Bosentan | 9 | (2.3) | 8 | (2.6) | 7 | (3.7) | 4 | (3.4) |
|  | Ambrisentan | 5 | (1.3) | 4 | (1.3) | 4 | (2.1) | 2 | (1.7) |
| NO | |  |  |  |  |  |  |  |  |
|  | Sildenafil | 10 | (2.6) | 7 | (2.3) | 2 | (1.0) | 1 | (0.9) |
|  | Tadalafil | 8 | (2.1) | 8 | (2.6) | 4 | (2.1) | 3 | (2.6) |
|  | Riociguat | 2 | (0.5) | 3 | (1.0) | 2 | (1.0) | 1 | (0.9) |
| PGI2 (po/inhaled) | |  |  |  |  |  |  |  |  |
|  | Beraprost | 32 | (8.3) | 27 | (8.8) | 10 | (5.2) | 5 | (4.3) |
|  | Iloprost | 1 | (0.3) | 0 | (0.0) | 0 | (0.0) | 0 | (0.0) |
|  | Selexipag | 1 | (0.3) | 0 | (0.0) | 0 | (0.0) | 0 | (0.0) |
| PGI2 (sc/iv) | |  |  |  |  |  |  |  |  |
|  | Epoprostenol | 1 | (0.3) | 0 | (0.0) | 0 | (0.0) | 0 | (0.0) |
| Combination therapy | |  |  |  |  |  |  |  |  |
| ERA + NO | |  |  |  |  |  |  |  |  |
|  | Macitentan + Tadalafil | 7 | (1.8) | 6 | (2.0) | 6 | (3.1) | 3 | (2.6) |
|  | Ambrisentan + Tadalafil | 5 | (1.3) | 7 | (2.3) | 3 | (1.6) | 3 | (2.6) |
|  | Bosentan + Sildenafil | 4 | (1.0) | 3 | (1.0) | 1 | (0.5) | 1 | (0.9) |
|  | Bosentan + Tadalafil | 4 | (1.0) | 3 | (1.0) | 1 | (0.5) | 1 | (0.9) |
|  | Macitentan + Riociguat | 2 | (0.5) | 3 | (1.0) | 1 | (0.5) | 1 | (0.9) |
|  | Macitentan + Sildenafil | 2 | (0.5) | 1 | (0.3) | 1 | (0.5) | 1 | (0.9) |
|  | Macitentan + Sildenafil + Riociguat | 2 | (0.5) | 0 | (0.0) | 0 | (0.0) | 0 | (0.0) |
|  | Ambrisentan + Sildenafil | 1 | (0.3) | 4 | (1.3) | 3 | (1.6) | 3 | (2.6) |
|  | Ambrisentan + Bosentan + Tadalafil | 0 | (0.0) | 1 | (0.3) | 0 | (0.0) | 0 | (0.0) |
|  | Bosentan + Tadalafil + Sildenafil | 0 | (0.0) | 0 | (0.0) | 1 | (0.5) | 0 | (0.0) |
|  | Macitentan + Ambrisentan + Tadalafil | 0 | (0.0) | 0 | (0.0) | 1 | (0.5) | 0 | (0.0) |
|  | Macitentan + Bosentan + Sildenafil | 0 | (0.0) | 0 | (0.0) | 2 | (1.0) | 0 | (0.0) |
|  | Macitentan + Bosentan + Tadalafil | 0 | (0.0) | 0 | (0.0) | 0 | (0.0) | 1 | (0.9) |
| ERA + PGI2 (po/inhaled) | |  |  |  |  |  |  |  |  |
|  | Bosentan + Beraprost | 7 | (1.8) | 4 | (1.3) | 4 | (2.1) | 2 | (1.7) |
|  | Ambrisentan + Beraprost | 0 | (0.0) | 1 | (0.3) | 1 | (0.5) | 1 | (0.9) |
|  | Macitentan + Beraprost | 0 | (0.0) | 1 | (0.3) | 0 | (0.0) | 0 | (0.0) |
| NO + PGI2 (po/inhaled) | |  |  |  |  |  |  |  |  |
|  | Sildenafil + Beraprost | 7 | (1.8) | 5 | (1.6) | 5 | (2.6) | 3 | (2.6) |
|  | Tadalafil + Selexipag | 3 | (0.8) | 2 | (0.7) | 2 | (1.0) | 0 | (0.0) |
|  | Riociguat + Iloprost | 1 | (0.3) | 0 | (0.0) | 0 | (0.0) | 0 | (0.0) |
|  | Tadalafil + Beraprost | 1 | (0.3) | 0 | (0.0) | 1 | (0.5) | 0 | (0.0) |
|  | Tadalafil + Sildenafil + Selexipag | 1 | (0.3) | 0 | (0.0) | 0 | (0.0) | 0 | (0.0) |
|  | Sildenafil + Selexipag | 0 | (0.0) | 1 | (0.3) | 0 | (0.0) | 0 | (0.0) |
|  | Tadalafil + Selexipag + Beraprost | 0 | (0.0) | 1 | (0.3) | 0 | (0.0) | 0 | (0.0) |
| NO + PGI2 (sc/iv) | |  |  |  |  |  |  |  |  |
|  | Sildenafil + Riociguat + Treprostinil | 0 | (0.0) | 1 | (0.3) | 0 | (0.0) | 0 | (0.0) |
| Triple | |  |  |  |  |  |  |  |  |
|  | Bosentan + Sildenafil + Beraprost | 4 | (1.0) | 4 | (1.3) | 2 | (1.0) | 1 | (0.9) |
|  | Macitentan + Tadalafil + Selexipag | 3 | (0.8) | 3 | (1.0) | 5 | (2.6) | 2 | (1.7) |
|  | Ambrisentan + Tadalafil + Beraprost | 2 | (0.5) | 0 | (0.0) | 0 | (0.0) | 0 | (0.0) |
|  | Ambrisentan + Sildenafil + Beraprost | 1 | (0.3) | 0 | (0.0) | 0 | (0.0) | 0 | (0.0) |
|  | Ambrisentan + Sildenafil + Epoprostenol | 1 | (0.3) | 1 | (0.3) | 1 | (0.5) | 0 | (0.0) |
|  | Ambrisentan + Tadalafil + Selexipag | 1 | (0.3) | 0 | (0.0) | 0 | (0.0) | 0 | (0.0) |
|  | Bosentan + Tadalafil + Beraprost | 1 | (0.3) | 3 | (1.0) | 1 | (0.5) | 1 | (0.9) |
|  | Bosentan + Tadalafil + Selexipag | 1 | (0.3) | 1 | (0.3) | 1 | (0.5) | 0 | (0.0) |
|  | Macitentan + Sildenafil + Beraprost | 1 | (0.3) | 0 | (0.0) | 0 | (0.0) | 0 | (0.0) |
|  | Macitentan + Tadalafil + Selexipag + Beraprost | 1 | (0.3) | 1 | (0.3) | 0 | (0.0) | 0 | (0.0) |
|  | Tadalafil + Selexipag + Epoprostenol | 1 | (0.3) | 0 | (0.0) | 0 | (0.0) | 0 | (0.0) |
|  | Ambrisentan + Bosentan + Sildenafil + Beraprost | 0 | (0.0) | 0 | (0.0) | 1 | (0.5) | 0 | (0.0) |
|  | Ambrisentan + Riociguat + Beraprost | 0 | (0.0) | 1 | (0.3) | 1 | (0.5) | 1 | (0.9) |
|  | Macitentan + Ambrisentan + Sildenafil + Epoprostenol | 0 | (0.0) | 0 | (0.0) | 0 | (0.0) | 1 | (0.9) |
|  | Macitentan + Ambrisentan + Tadalafil + Selexipag | 0 | (0.0) | 1 | (0.3) | 0 | (0.0) | 0 | (0.0) |
|  | Macitentan + Riociguat + Iloprost | 0 | (0.0) | 0 | (0.0) | 1 | (0.5) | 1 | (0.9) |
|  | Macitentan + Tadalafil + Beraprost | 0 | (0.0) | 1 | (0.3) | 0 | (0.0) | 0 | (0.0) |
|  | Macitentan + Tadalafil + Selexipag + Treprostinil | 0 | (0.0) | 0 | (0.0) | 0 | (0.0) | 1 | (0.9) |
| Abbreviations: PoPH, portopulmonary hypertension; ERA, endothelin receptor antagonist; NO, nitric oxide; PGI2 (po/inhaled), prostacyclin (oral/inhaled); PGI2 (sc/iv), prostacyclin (subcutaneous/intravenous). | | | | | | | | | |

**
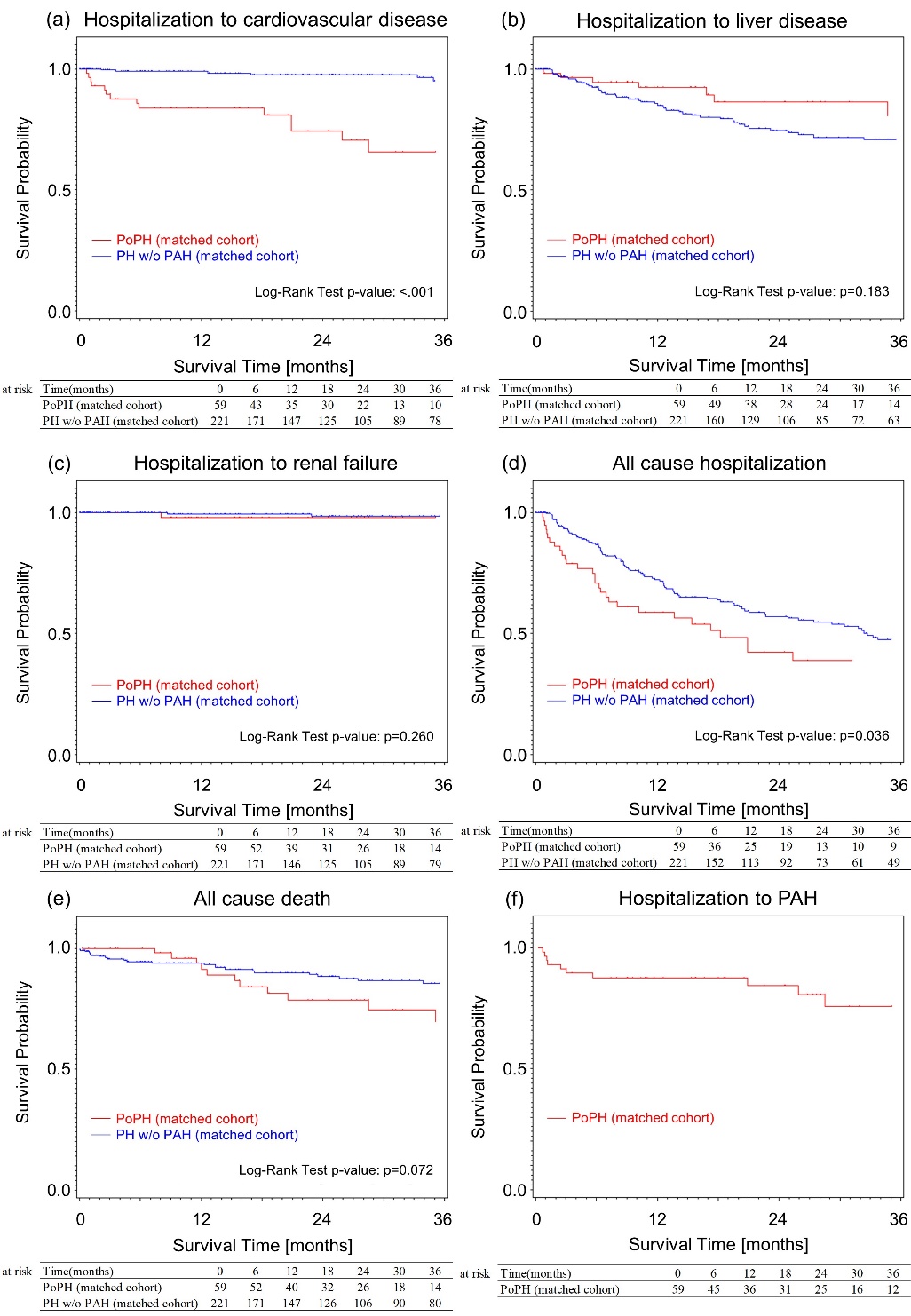
**

**Figure S1-A Time to event survival analysis (Kaplan Meier plot) (Child-Pugh class A)**

(a) The time to emergency hospitalization related to cardiovascular disease was significantly different between PoPH group and portal hypertension w/o PAH group (*p* <0.001 by log-rank test). (d) The median of time to all cause emergency hospitalization was 18.2 months for PoPH group and 32.7 months for portal hypertension w/o PAH group (*p* = 0.036 by log-rank test).


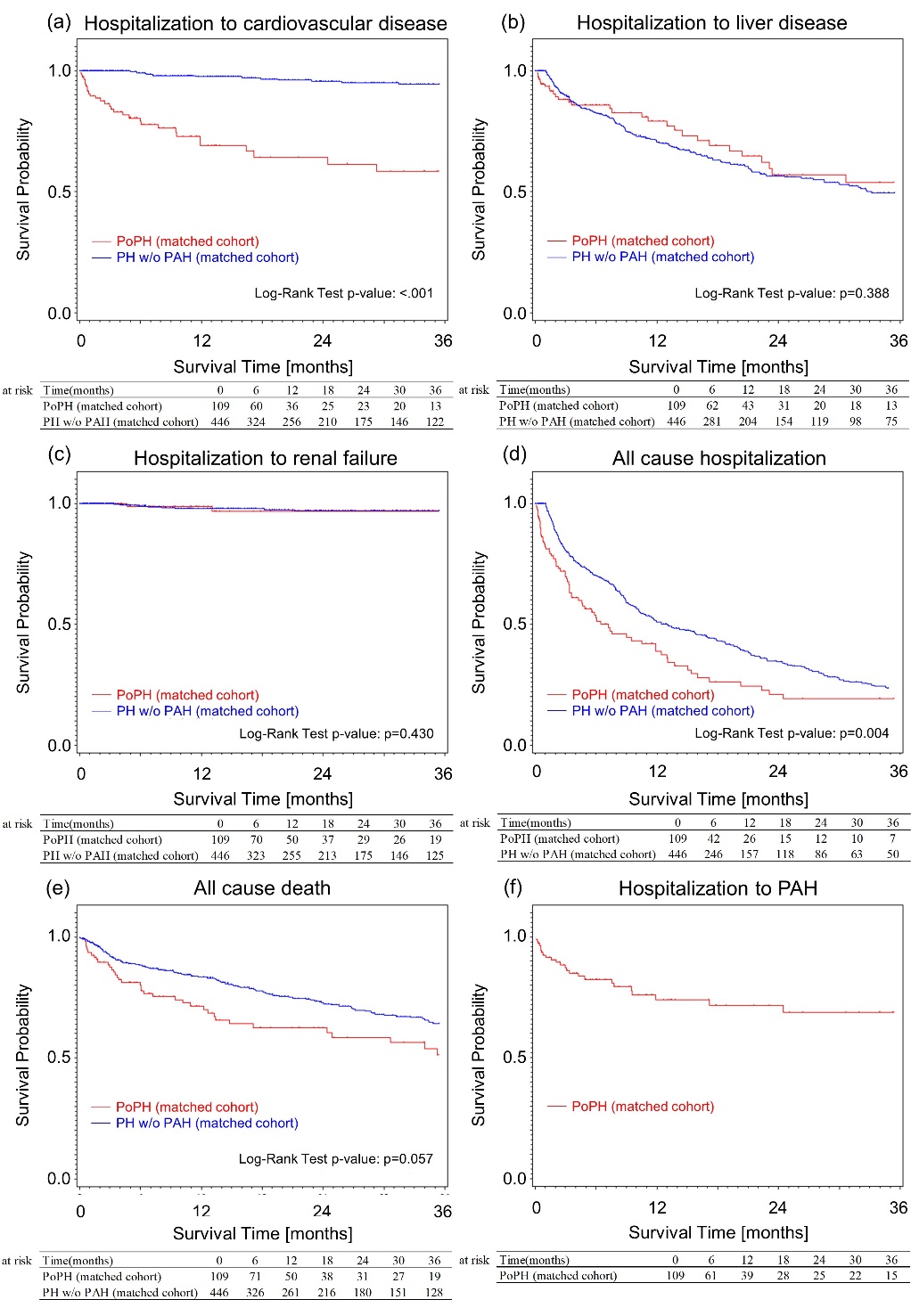


**Figure S1-B Time to event survival analysis (Kaplan Meier plot) (Child-Pugh class B)**

(a) The median of time to emergency hospitalization attributable to cardiovascular diseases was 54.1 months for PoPH group (*p* <0.001 by log-rank test). (d)The median of time to all cause emergency hospitalization was 6.5 months for PoPH group and 13.0 months for portal hypertension w/o PAH group (*p* = 0.004 by log-rank test).


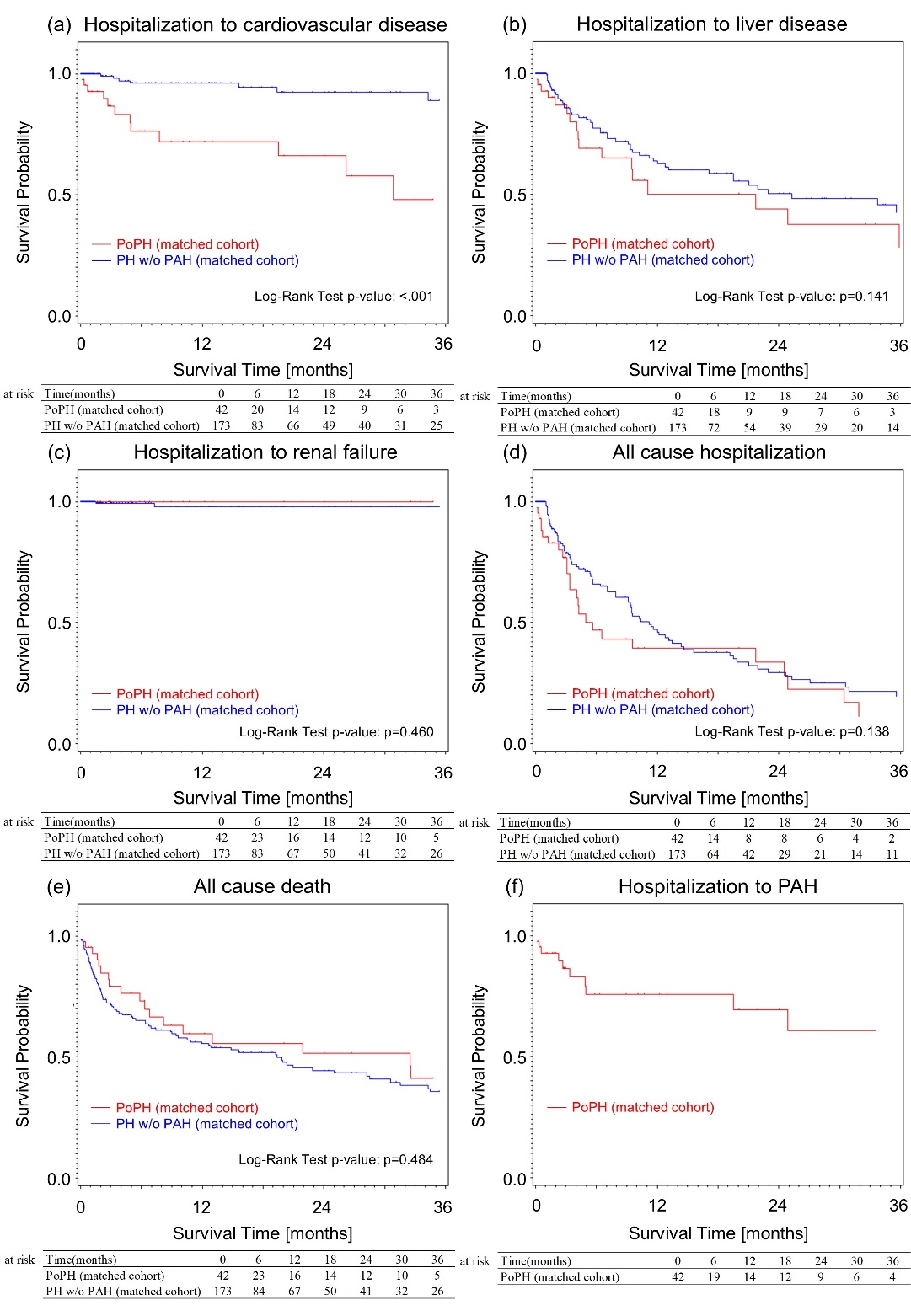


**Figure S1-C Time to event survival analysis (Kaplan Meier plot) (Child-Pugh class C)**

(a) The median of time to emergency hospitalization attributable to cardiovascular diseases was 30.8 months for PoPH group and could not be derived for portal hypertension w/o PAH group (*p* <0.001 by log-rank test).
